# A multicenter randomized trial comparing rabeprazole and itopride in patients with functional dyspepsia in Japan: the NAGOYA study

**DOI:** 10.3164/jcbn.16-106

**Published:** 2017-02-24

**Authors:** Takeshi Kamiya, Michiko Shikano, Eiji Kubota, Tsutomu Mizoshita, Tsuneya Wada, Satoshi Tanida, Hiromi Kataoka, Hiroshi Adachi, Makoto Hirako, Noriaki Okuda, Takashi Joh

**Affiliations:** 1 Department of Medical Innovation, Nagoya City University Graduate School of Medical Sciences, Kawasumi 1, Mizuho-cho, Mizuho-ku, Nagoya 467-8601, Japan; 2 Department of Gastroenterology and Metabolism, Nagoya City University Graduate School of Medical Sciences, Kawasumi 1, Mizuho-cho, Mizuho-ku, Nagoya 467-8601, Japan; 3 Public Health Center, Okazaki City Medical Association, Tatsumi nishi 1-9-1, Okazaki, Aichi 444-0875, Japan; 4 Adachi Clinic, Yagotoyama 220, Tenpaku-ku, Nagoya 468-0077, Japan; 5 Fuji Hospital, Nishiyashiki 137-1, Ushida-cho, Chiryu, Aichi 472-0007, Japan; 6 Okuda Naika Clinic, Hinata-cho 2-9-3, Mizuho-ku, Nagoya 467-0047, Japan

**Keywords:** functional dyspepsia, itopride, rabeprazole, epigastric pain syndrome, postprandial distress syndrome

## Abstract

The aims of this study were to compare the therapeutic effects of a proton pump inhibitor (PPI), rabeprazole (RPZ), and a prokinetic agent, itopride (ITO), and to investigate the role of PPI in the treatment strategy for Japanese functional dyspepsia (FD) patients. We randomly assigned 134 patients diagnosed by Rome III criteria to 4 weeks treatment with RPZ 10 mg/day (*n* = 69) or ITO 150 mg/day (*n* = 65). Dyspeptic symptoms were evaluated using FD scores at baseline and after 1, 2 and 4 weeks of treatment. We also divided subjects into predominantly epigastric pain syndrome (EPS) or postprandial distress syndrome (PDS), and evaluated the efficacy of RPZ and ITO respectively. RPZ showed a significant decrease in the Rate of Change (RC) in FD score within 1 week, which was maintained until after 4 weeks, with RPZ a significant effect compared with ITO at all evaluation points. In addition, RPZ showed a significant decrease in FD score in subjects with both EPS and PDS, whereas a significant decrease in the RC with ITO was only shown in those with predominant PDS. Acid-suppressive therapy with RPZ is useful for PDS as well EPS in Japanese FD patients (UMIN Clinical Trials Registry number: UMIN 000013962).

## Introduction

Functional dyspepsia (FD) is a common clinical syndrome characterized by persistent or recurrent upper abdominal symptoms, such as epigastric pain, postprandial fullness and early satiety, in the absence of any organic disease that is likely to explain the symptoms. In the Rome III criteria, FD is subdivided into two diagnostic categories: 1) postprandial distress syndrome (PDS), characterized by postprandial fullness and early satiation; and 2) epigastric pain syndrome (EPS), characterized by epigastric pain and burning.^(1)^ Several studies in Western countries have reported the prevalence of FD to be between 10% and 20% of the adult population.^(2–4)^ In Japan, the number of patients with FD is increasing, and FD accounts for approximately 45% of outpatients presenting with upper abdominal symptoms.^(5)^ It is widely accepted that a variety of factors including abnormal gastric emptying, visceral hypersensitivity and autonomic nervous system disorder contribute to the development of FD,^(6–8)^ and there is considerable heterogeneity in symptom patterns.^(9)^ Although a variety of pharmacological agents are used in clinical practice, including suppressors of gastric acid secretion, prokinetic agents, and antidepressants, the treatment of FD remains a considerable challenge. To improve clinical results, we need therapeutic approaches directed at the underlying pathophysiology.

In general, the level of acid secretion in the Japanese population is thought to be lower than in Western people. Prokinetic agents and H2 receptor antagonists are widely used to treat Japanese FD patients.^(10)^ A comparison of efficacy between proton pump inhibitors (PPIs) and prokinetics in Japanese FD patients has yet to be performed.

The aim of this study was to investigate and compare the therapeutic effects of a PPI, rabeprazole (RPZ), and itopride (ITO), a dopamine D2 agonist with acetylcholinesterase activity (i.e., prokinetic agent), in Japanese FD patients.

## Methods

### Study design

This randomized open-label trial (UMIN Clinical Trials Registry number: UMIN 000013962 http://www/umin.ac.jp/ctr/) was conducted at 4 institutions in Japan. The study protocol was approved by the Institutional Review Board of Nagoya City University Graduate School of Medical Sciences on behalf of each participating institution. This study was performed in accordance with the Declaration of Helsinki.

### Study population

Subjects aged over 20 years with dyspeptic symptoms such as upper abdominal pain and postprandial fullness, in whom organic disease had been excluded by upper endoscopy and who met the Rome III criteria for FD, were included. All subjects gave written informed consent to participate.

The exclusion criteria were: 1) history of gastrectomy; 2) organic brain disease, schizophrenia, or a predisposition to schizophrenia; 3) alcoholism or other substance abuse disorder; 4) serious hormonal imbalance (e.g., hyperthyroidism); 5) serious heart, liver, kidney or hematopoietic disease; 6) history of hypersensitivity to either of the test agents; 7) pregnant or lactating women, women who might fall pregnant, or hoped to become pregnant, during the study period; and 8) other potential subjects deemed unsuitable by their treating physician. Use of concomitant medications that might interact with the test agents, or affect evaluation of their pharmaceutical effects (e.g., gastroprokinetic agents, antiulcer agents, and anticholinergic agents), was not allowed. If medications of this nature were used prior to study entry, there was a washout period of at least 7 days before study commencement. Concomitant medications were allowed for pre-existing conditions if the investigating physician considered their continuation would not affect evaluation of the test agents, with dosage reduction or discontinuation to be avoided during the study period.

### Symptom assessment

**Fig. 1:**
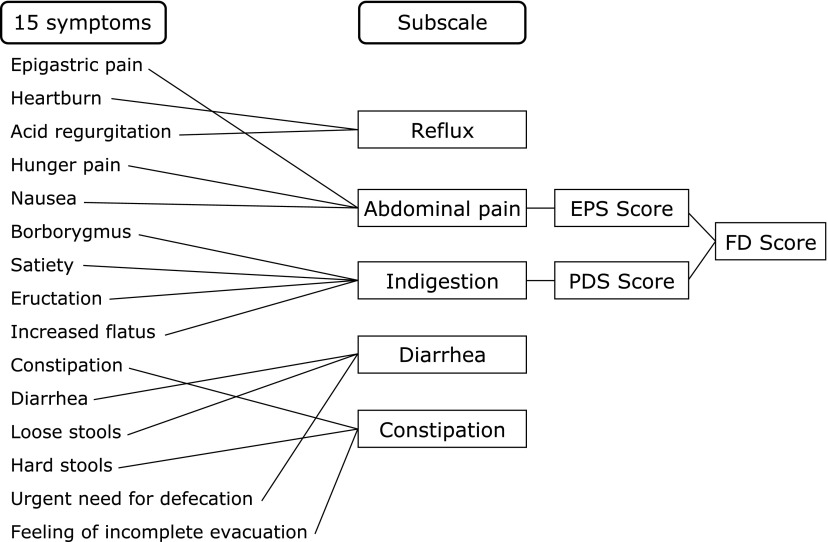
Meaning of FD scores. GSRS, Gastrointestinal Symptom Rating Scale; FD, functional dyspepsia; EPS, epigastric pain syndrome; PDS, postprandial distress syndrome.

Eligible subjects were randomly allocated to 4 weeks treatment with RPZ 10 mg once-daily or ITO 50 mg three times -daily in a 1:1 ratio using computerized random numbers. Subjects were asked to evaluate their own symptoms pretreatment and after 1, 2 and 4 weeks’ treatment by filling in the Gastrointestinal Symptom Rating Scale (GSRS).^(11,12)^ The GSRS includes 15 items related to general gastrointestinal symptoms, and uses a 7-point Likert scale ranging from “not at all bothered” to “unbearably bothered”. The 15 items were combined into 5 symptom clusters: reflux, abdominal pain, indigestion, diarrhea and constipation. In case of FD, the abdominal pain and indigestion scores are considered to represent the EPS and PDS scores, respectively. The FD score was defined as the sum of the EPS and PDS scores in this study (Fig. 1).^(12,13)^ The maximum possible FD score was 49. We checked compliance with the test agents at each attendance, and withdrew subjects from the study if it fell below 80%.

### Study endpoints

The primary endpoint was the Rate of Change (RC, %) in the FD score in the 4 weeks of treatment in a per-protocol (PP) population analysis. The secondary endpoints were the RC in the EPS score and that in the PDS score between pretreatment (100%) and each time-point. We also conducted a sub-analysis of EPS and PDS based on the Rome III criteria.^(1)^

### Sample size

Sub-analysis of subjects with epigastric pain as their main symptom in a study with *Helicobacter pylori* (HP) negative uninvestigated dyspeptic patients showed symptomatic improvement in 47% of subjects administered omeprazole, and 23% of those given the prokinetic agent cisapride.^(14)^ Based on a two-sided level of significance of 5%, and a statistical power of 80% (statistical method: χ^2^ test), we calculated a required subject number of 60.7 subjects per group. Allowing for dropouts, we aimed for 65 subjects per group, for a total of 130.

### Statistical analysis

Data are given as mean ± SD. We used Fisher’s exact probability test and the Mann-Whitney *U* test for comparisons between groups. We used the one-way ANOVA test for comparisons of pre- and post-treatment scores. *P* values less than 0.05 were considered statistically significant. Statistical analyses were performed using SPSS ver. 19 (Chicago, IL).

## Results

**Fig. 2:**
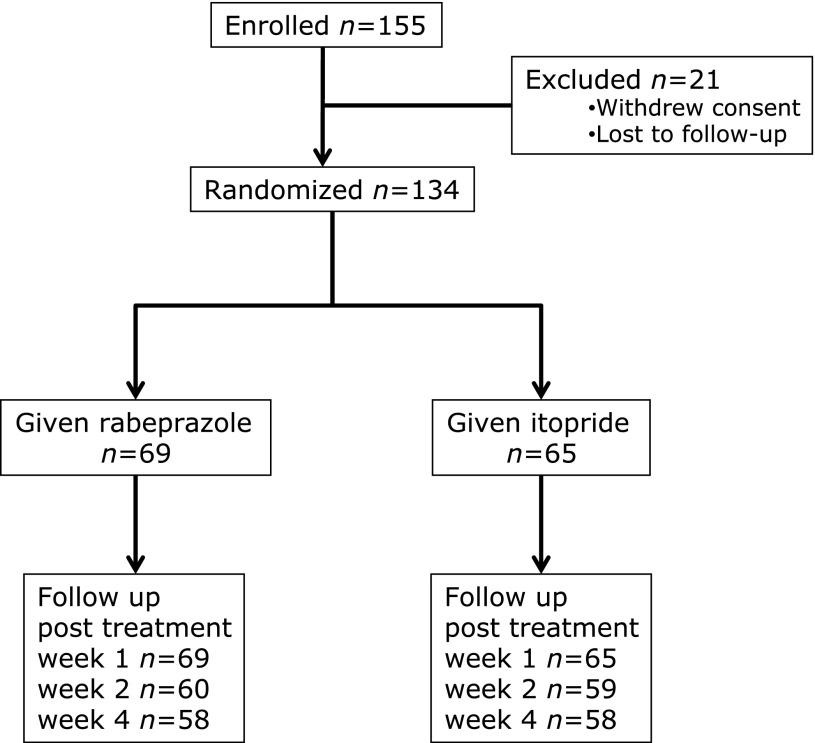
Flow diagram of a multicenter trial comparing functional dyspepsia symptoms between subjects treated with rabeprazole or itopride.

We enrolled 155 subjects who presented to the participating institutions between February 2007 and December 2008. After exclusion of 21 potential subjects from whom informed consent could not be obtained because of failure to keep appointments or other reasons, 134 subjects (male/female 89/45, average age 52.4 ± 14.8 years) were assigned to receive either RPZ (*n* = 69) or ITO (*n* = 65) for 4 weeks (Fig. 2). Table 1 shows the subjects’ baseline clinical characteristics in the PP population. No significant differences were seen between treatment groups in pretreatment age, sex, height, body mass index (BMI), HP status (serum anti-HP antibody titers), duration of symptoms, pre-entry medications, or GSRS and FD scores.

### Primary endpoint

**Fig. 3:**
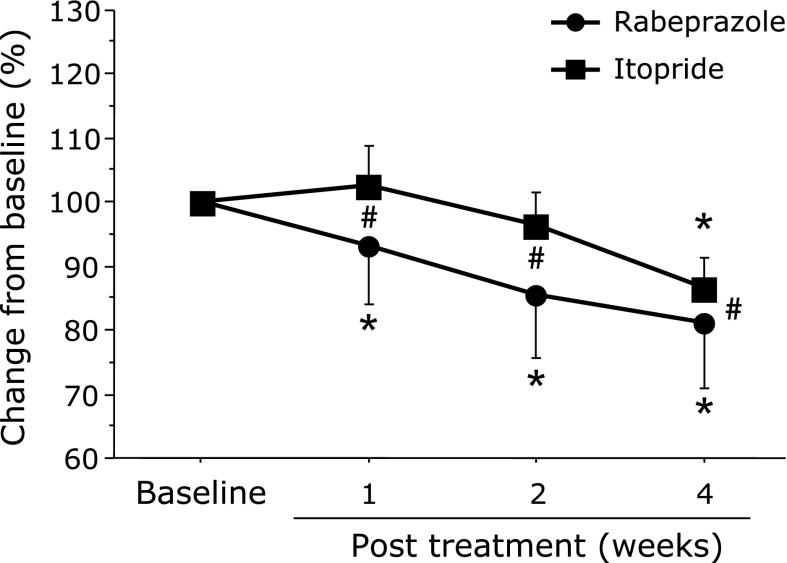
Comparison of percentage change in FD scores between treatment groups from baseline to post treatment. FD, functional dyspepsia; RPZ, rabeprazole; ITO, itopride. *p<0.05, comparison with pretreatment for each group. ^#^*p*<0.05, comparison between groups. Data are given as mean ± SD.

RC in FD score (%): The RPZ group showed a significant decrease in the RC in FD scores within 1 week (93.2 ± 63.2%, *p* = 0.005), which was maintained until after 4 weeks of treatment. A significant decrease in the FD score was seen after 4 weeks of treatment with ITO (86.5 ± 33.7%, *p* = 0.0014). In addition, the RPZ group showed a significant effect compared with the ITO group at all time points (1 week: *p* = 0.0367, 2 weeks: *p* = 0.0029, 4 weeks: *p* = 0.0491) (Fig. 3).

### Secondary endpoints

**Fig. 4:**
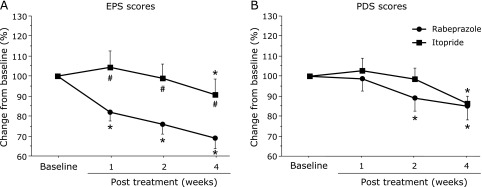
Comparison of percentage change in EPS (A) and PDS (B) scores between treatment groups from baseline to post treatment. FD, functional dyspepsia; RPZ, rabeprazole; ITO, itopride; EPS, epigastric pain syndrome; PDS, postprandial distress syndrome. *p<0.05, comparison with pre treatment for each group. ^#^*p*<0.05, comparison between groups. Data are given as mean ± SD.

RC in EPS score (%): The RPZ group showed a significant decrease in EPS score after 1 week of treatment (82.0 ± 31.3%, *p* = 0.0003), which was maintained until after 4 weeks of treatment (68.9 ± 34.9%, *p*<0.0001). A significant decrease in the EPS scores was only seen after 1 week of treatment with ITO (90.5 ± 53.1%, *p* = 0.0479). Intergroup comparison showed significantly greater RC with RPZ than with ITO at each timepoint (1 week: *p* = 0.0194, 2 weeks: *p* = 0.0078, 4 weeks: *p* = 0.0273) (Fig. 4, left side).

RC in PDS score (%): A significant decrease in PDS scores was seen after 2 weeks of treatment with RPZ (89.0 ± 44.8%, *p* = 0.0032), which was maintained until after 4 weeks of treatment (84.9 ± 46.0%, *p* = 0.0007). The ITO group showed a significant decrease in PDS scores after 4 weeks of treatment (96.2 ± 24.5%, *p* = 0.0008). No significant differences were observed between the RPZ and ITO groups at any time-point (Fig. 4, right side).

### Sub-analysis assessment according to EPS/PDS

**Fig. 5:**
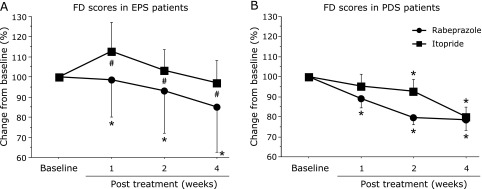
Comparison of percentage change in FD scores between treatment groups from baseline to post treatment for subjects with EPS (A) and PDS (B). FD, functional dyspepsia; RPZ, rabeprazole; ITO, itopride; EPS, epigastric pain syndrome; PDS, postprandial distress syndrome. *p<0.05, comparison with baseline for each group. ^#^*p*<0.05, comparison between groups. Data are given as mean ± SD.

RC in FD score in patients with EPS (%): A significant decrease in FD scores was observed after 1 week of treatment with RPZ in subjects with predominant EPS (98.7 ± 90.2%, *p* = 0.0102), which was maintained until after 4 weeks of treatment (85.2 ± 101.6%, *p* = 0.0057). In the ITO group, no significant RC was seen in the FD scores of subjects with EPS (4 weeks: 97.3 ± 44.1%, *p* = 0.4074). Intergroup comparison demonstrated significantly greater RC in the RPZ EPS subgroup than in the ITO EPS subgroup at each time-point (1 week: *p* = 0.0431, 2 weeks: *p* = 0.0194, 4 weeks: *p* = 0.0100) (Fig. 5, left side).

RC in FD score in patients with PDS (%): The RPZ group showed a significant decrease in FD scores after 1 week of treatment in patients with predominant PDS (1 week: 78.5 ± 25.6%, *p* = 0.0379, 2 weeks: 79.6 ± 18.7%, *p* = 0.0002, respectively), which was maintained until after 4 weeks of treatment (78.5 ± 25.6%, *p* = 0.0017). A significant decrease in RC was observed after 2 and 4 weeks of treatment with ITO (2 weeks; 92.7 ± 30.3%, *p* = 0.0455, 4 weeks: 79.9 ± 23.6%, *p* = 0.0011). No significant differences in FD scores were seen between the RPZ PDS and ITO PDS subgroups at any evaluation point (Fig. 5, right side).

### Adverse events

There were no adverse effects in either group.

## Discussion

In this study, we demonstrated that acid-suppressive therapy with RPZ is useful for Japanese FD patients, as reported in Western countries. This study is the first randomized trial of a PPI and prokinetic agent in the treatment of Japanese FD patients. Many trials in Western countries have demonstrated the efficacy of PPIs,^(14–16)^ and meta-analyses have confirmed the benefits of PPI therapy. Acid suppression with a PPI is recommended as first-line treatment, especially in HP-negative patients with FD.^(17)^ In Japan, published trials, such as the Samurai study, have investigated the efficacy of PPI therapy in patients with FD,^(18)^ although famotidine (H_2_-receptor antagonist),^(10)^ tandospiron (partial agonist of 5-HT 1A),^(19)^ and Rikunshito (a herbal medicine) have been shown to improve dyspeptic symptoms in Japanese FD patients.^(20)^ The present study showed that FD scores significantly improved after 1 week in patients administered RPZ, indicating a rapid onset of effect by the PPI in Japanese FD patients in the overall analysis.

RPZ therapy provided significant improvements in the FD scores of both EPS- and PDS- predominant patients in this study. The Rome II criteria recommend the use of acid- suppressing agents, including PPIs, for patients with ulcer-like dyspepsia,^(15)^ and prokinetic agents for those with dysmotility-like dyspepsia.^(21)^ Currently, there is a lack of evidence on treatment approaches to the EPS and PDS categories, newly defined by the Rome III committee. Nevertheless, based on the Rome III criteria, PPIs are generally the first-line therapy for EPS, and prokinetic agents for PDS.^(22)^ In the present study, the PPI demonstrated significant RC in both EPS scores in the overall analysis and FD scores in subjects with EPS, within 1 week. Furthermore, RPZ showed significant efficacy compared with ITO in the EPS and FD scores at all time points. These results suggest that increased exposure of the gastroduodenal mucosa to acid plays an important role in the pathogenesis of EPS.

In the ITO group, significant RC was only shown with PDS patients. Prokinetic agents are widely prescribed for patients with FD. Although a meta-analysis showed cisapride was more effective than placebo,^(23)^ its use is now severely restricted because of cardiac problems. ITO has shown benefit in some trials. It was reported as effective in the treatment of FD in a phase II trial,^(24)^ but no superiority to placebo was seen in the phase III trial.^(25)^ Prokinetic agents are thought to ameliorate disturbances of gastrointestinal motility, such as delayed gastric emptying.^(26,27)^ Disturbed gastric motility has been proposed as a key mechanism underlying symptom generation in patients with PDS. The results of these earlier studies agree with our results that ITO was useful for PDS and had no discernible effect on EPS.

Additional interesting results in our study are that both the PPIs and prokinetic agent were useful for PDS. Significant RC in the PDS score was shown to a similar degree after treatment with RPZ and ITO. In subjects with predominant PDS, the FD score improved significantly in both the RPZ and ITO groups. In addition, there were no significant differences in the FD or PDS scores between treatment groups. Improvement in PDS-like symptoms, including postprandial fullness, was also observed with acid suppression by the PPI, suggesting that gastroduodenal acidity is related to the underlying mechanism of symptom generation in some patients with PDS-predominant FD. In recent years, some studies have reported that acid infusion into the stomach or duodenum induces dyspeptic symptoms such as bloating,^(28,29)^ in both healthy volunteers and patients with FD. Furthermore, gastric or duodenal acidity may be related to delayed gastric emptying, impaired gastric accommodation and increased gastric or duodenal sensitivity.^(30,31)^ Not only gastric motility, but also gastroduodenal acidity, may play a role in the dyspeptic symptoms in PDS patients.

One characteristic of Japanese FD patients is the high prevalence of HP infection compared with Western patients.^(32)^ Pathophysiologically, FD is a heterogenous disorder, and several mechanisms have been suggested to play a role in the etiology of dyspeptic symptoms. HP infection has been proposed as a possible mechanism related to symptom generation. Atrophic gastritis following HP infection may lead to delayed gastric emptying or gastric hypersensitivity.^(33)^ However, the efficacy of HP eradication in improving dyspeptic symptoms is still controversial.^(34–36)^ Further studies will be needed to clarify the relationship between HP infection and dyspeptic symptoms.

In conclusion, we demonstrated that acid-suppressive therapy with a PPI is also useful in Japanese FD patients, similar to results from Western countries. RPZ was effective for both PDS and EPS, whereas ITO was effective for PDS. These results provide supportive evidence for the development of new management strategies in the treatment of Japanese FD patients.

## Author Contributions

Takeshi Kamiya, guarantor of the article, specific author contributions, design of the study, writing the protocol, enrolling patients, conducting the study and writing the manuscript; Michiko Shikano, writing the protocol and performing statistical analyses; Tsutomu Mizoshita, Tsuneya Wada, Satoshi Tanida, Hiromi Kataoka, Hiroshi Adachi, Makoto Hirako and Noriaki Okuda, enrolling patients and conducting the study; Takashi Joh, design of the study and reviewing the manuscript.

All authors have approved the submitted manuscript.

## Figures and Tables

**Table 1:** Clinical characteristics of Japanese subjects with functional dyspepsia

	rabeprazole (*n* = 69)	itopride (*n* = 65)
Age	51.2 ± 15.2 (24–79)	53.6 ± 14.2 (19–79)
Gender (male/female)	27/42	18/47
Height (cm)	160.3 ± 7.8	157.9 ± 9.0
Weight (kg)	53.0 ± 11.4	52.6 ± 11.7
Body mass index (kg/m^2^)	20.5 ± 3.6	21.0 ± 3.8
*Helicobacter pylori* status (positive/negative/unknown)	23/35/11	29/26/10
Duration of symptoms (years)	4.5 ± 8.9 (0.5–55.0)	4.5 ± 10.0 (0.5–55.0)
Medication before entry (YES/NO)	32/37	36/38
Type of Medicine		
Proton pump inhibitor	0	4
Histamine 2 receptor antagonist	12	16
Prokinetic agent	3	3
Other (gastro-protective, etc.)	17	28
FD subgroup (EPS/PDS/overlap)	26/35/4	33/33/3
GSRS total score	2.6 ± 1.0	2.6 ± 1.0
Subscale scores		
Reflux	2.5 ± 1.1	2.7 ± 1.5
Abdominal pain	3.1 ± 1.4	2.7 ± 1.3
Indigestion	2.8 ± 1.2	2.6 ± 1.2
Diarrhoea	2.3 ± 1.3	2.3 ± 1.4
Constipation	2.5 ± 1.5	2.7 ± 1.2
FD score	3.0 ± 1.3	2.7 ± 1.3

No significant difference between treatment groups. The FD score was the sum of the EPS score and the PDS score.

## Abbreviations

EPS: epigastric pain syndrome
FD: functional dyspepsia
GSRS: Gastrointestinal Symptom Rating Scale
HP: *Helicobacter pylori*
ITO: itopride
PDS: postprandial distress syndrome
PPI: proton pump inhibitor
RC: rate of change
RPZ: rabeprazole
